# A two-stage decision-support approach for improving sustainable last-mile cold chain logistics operations of COVID-19 vaccines

**DOI:** 10.1007/s10479-022-04906-x

**Published:** 2022-08-21

**Authors:** Eugenia Ama Andoh, Hao Yu

**Affiliations:** Department of Industrial Engineering, UiT The Arctic University of Norway, Lodve Langesgate 2, 8514 Narvik, Norway

**Keywords:** Sustainable logistics, Green transportation, Vaccine logistics, Cold chain logistics, COVID-19 vaccines, Simulation

## Abstract

The COVID-19 pandemic has become a global health and humanitarian crisis that catastrophically affects many industries. To control the disease spread and restore normal lives, mass vaccination is considered the most effective way. However, the sustainable last-mile cold chain logistics operations of COVID-19 vaccines is a complex short-term planning problem that faces many practical challenges, e.g., low-temperature storage and transportation, supply uncertainty at the early stage, etc. To tackle these challenges, a two-stage decision-support approach is proposed in this paper, which integrates both route optimization and advanced simulation to improve the sustainable performance of last-mile vaccine cold chain logistics operations. Through a real-world case study in Norway during December 2020 and March 2021, the analytical results revealed that the logistics network structure, fleet size, and the composition of heterogeneous vehicles might yield significant impacts on the service level, transportation cost, and CO_2_ emissions of last-mile vaccine cold chain logistics operations.

## Introduction

The novel coronavirus disease (COVID-19) has become one of the most catastrophic events in recent human history, which dramatically influences the world’s economy, overwhelms the healthcare systems, disrupts the global supply chains (Ivanov, 2020), and alters people’s lifestyles. As of May 9th, 2021, over 150 million confirmed cases of COVID-19 have been recorded with more than 3.2 million associated deaths, according to the World Health Organization (WHO) (WHO Coronavirus (COVID-19) Dashboard). Mass vaccination is considered the most effective way to control the disease spread (Jiang et al., 2021) and minimize the associated deaths, which can help to restore normal lives. The WHO estimates that, by mass vaccination and immunization, approximately two to three million deaths can be prevented. In December 2020, the COVID-19 vaccine from Pfizer/BioNTech became the first one that passed the phase 3 trial and was approved for mass vaccination in order to flatten the curve of the second wave of COVID-19 outbreaks. In addition, several other COVID-19 vaccines, e.g., Moderna, AstraZeneca, etc., have also been approved for use in mass vaccination programs in several countries.

Even though the development of effective vaccines for COVID-19 has become unprecedentedly accelerated, there are still significant challenges related to the manufacturing, allocation, and logistics in order to fulfill the large global demands for mass vaccination (Kim et al., 2021), especially in the early vaccination stage. To ensure the global access and equitable allocation of COVID-19 vaccines, the WHO in cooperation with several large manufacturers and many countries launches the COVAX program, which aims particularly at helping the vulnerable and low-income countries to get COVID-19 vaccines. While the COVAX program improves the equitable global allocation of COVID-19 vaccines, the cold chain logistics infrastructures and operations are of paramount importance for the effective and efficient last-mile vaccine distribution at national and regional levels. However, the balance of several influencing factors, e.g., stringent temperature requirements and timeliness of vaccine delivery, etc., poses significant logistics challenges for last-mile cold chain logistics operations to distribute COVID-19 vaccines from the warehouse to the locations of administration (Kartoglu et al., 2020). For instance, the temperature requirement of the Pfizer-BioNTech's COVID-19 vaccine is at − 70 °C, which requires specialized equipment during the transportation and storage (https://www.pfizer.com/news/hot-topics/covid_19_vaccine_u_s_distribution_fact_sheet).

Compared with other cold chain logistics systems, the most significant challenge of the COVID-19 vaccine distribution is related to supply uncertainty at the early vaccination stage. This uncertainty yields bullwhip effects and delays the downstream distribution to the local vaccination centers. Because of this reason, the planning and scheduling of the local vaccination are drastically affected (Lee & Chen, 2021), and a large number of vaccines are wasted due to ineffective vaccination planning. A recent report by Mahase (2020) shows that many GPs in the UK are frustrated with the logistics problem and delayed delivery of the Pfizer-BioNTech’s COVID-19 vaccines, which result in a large number of booking cancellations and rescheduling of the vaccination. Furthermore, the sustainability issues of cold chain logistics operations have also been highlighted (Jiang et al., 2021; Klemeš et al., 2021), which, for example, may cause large energy consumption and environmental impacts to maintain the temperature requirement during transportation and storage. The most significant challenge of a sustainable logistics system for COVID-19 vaccines is to balance economic, environmental, and societal sustainability. In many countries, e.g., Norway, the amount of the COVID-19 vaccines received at the initial stage is very small, and these vaccines need to be fairly allocated and responsively distributed to a large number of geographically dispersed municipalities. On the one hand, this improves equity and societal sustainability. However, on the other hand, more resources are needed to maintain a high level of responsiveness of last-mile logistics operations, which, in consequence, leads to a reduced resource utilization, increased transportation costs, and more CO_2_ emissions, due to the loss of economy of scale.

Thus, in this paper, the sustainable last-mile cold chain logistics operations for effective and efficient COVID-19 vaccine distribution are focused on. Through the development of a quantitative analysis combining both route optimization and advanced simulation, we aim at answering the following three research questions:

- *RQ1* How can optimization models and advanced simulation, e.g., discrete event simulation, be jointly used for providing timely decision support of last-mile vaccine logistics operations?
- *RQ2* What are the most important influencing factors for sustainable last-mile cold chain logistics operations of COVID-19 vaccines?
- *RQ3* How do these influencing factors interact with the societal, economic, and environmental sustainability dimensions in the COVID-19 vaccine distribution.

The rest of the paper is structured as follows. Section 2 provides an overview of relevant literature related to sustainable vaccine logistics and supply chains. Based on the COVID-19 vaccine cold chain logistics system in Norway, the main challenges are discussed in Sect. 3. Section 4 proposes a two-stage decision-support approach for improving the sustainable last-mile cold chain logistics operations of COVID-19 vaccines. Section 5 presents a real-world application of the proposed method. Section 6 discusses the analytical results and generalizes some key managerial implications. Finally, the conclusions and laminations are given in the last section.

## Literature review

To tackle the logistics and supply chain challenges related to the overwhelmed healthcare systems during the COVID-19 pandemic, several studies have been conducted. New multi-objective optimization models combined with improved metaheuristics and simulation have been developed to provide better decision support for temporary medical centers (Ghasemi et al., 2021) and healthcare supply chains (Shirazi et al., 2021). In this regard, Goodarzian et al. (2021b) developed an integrated framework with both simulation and optimization for network design of sustainable medical supply chains during the pandemic, where several metaheuristics were tested. Goodarzian et al. (2021a) investigated a multi-product multi-objective model with stochastic chance-constraints for designing a sustainable and resilient healthcare network under uncertainties. Yu et al. (2020b) and Tirkolaee et al. (2021) optimized the reverse logistics systems for managing the sharply increased medical waste during the COVID-19 pandemic.

The global distribution of COVID-19 vaccines is one of the most significant logistics challenges, which requires an effective and coordinated vaccine supply chain. A vaccine supply chain is a complex network comprised of facilities and infrastructures, transport links and vehicles, specialized equipment, and personnel from the production to the administration at the end users (Lee et al., 2016). Managing a vaccine supply chain requires advanced planning and well-organized implementation at various stages to properly deal with uncertainties (Lemmens et al., 2016). At the global level, one of the most important goals of the vaccine supply chain is to ensure a fair allocation of vaccines to the targeted populations, and this requires better communications between the decision-makers and a large number of stakeholders (Lee & Haidari, 2017). Research has revealed that the enhanced stakeholder coordination provides a better chance for improving the fairness and responsiveness of a vaccine supply chain (Brison & Let al.,lec, 2017). Besides, due to the lack of the proper consideration of stakeholders’ involvement, the use of many analytical models in solving real-world problems is still very limited (De Boeck et al., 2019).

An effective and efficient vaccine supply chain needs to fulfill the customer demands at the right time and by the right quality and quantity of vaccines, while simultaneously, maintaining cost-effectiveness and environmentally friendliness. However, operating a vaccine supply chain needs to properly deal with many uncertainties, e.g., production fluctuation at the initial stage, facility or route disruptions, etc., which significantly hinders the viability and sustainability. Several studies suggest that the integration of a vaccine supply chain into some existing supply chains of other pharmaceutical products is an effective solution to improve the economic, environmental, and societal sustainability, particularly when the integration is done at the downstream distribution stage (Privett & Gonsalvez, 2014). Another important factor that affects a sustainable vaccine supply chain is the human factor (Ashok et al., 2017). For example, one of the most important causes of vaccine damage during transportation and storage is related to the incorrect operations by the workers with inadequate knowledge and training (Lloyd & Cheyne, 2017).

There are, in general, four main stages in a vaccine supply chain, namely, (1) the vaccine development and clinical trial, (2) the production of vaccines, (3) the formulation of the allocation strategy, and (4) the distribution to the vaccination centers (Rastegar et al., 2021). During the COVID-19 pandemic, the vaccine development and clinical trial have been unprecedentedly accelerated, but there are significant challenges for vaccine production and distribution due to the large global demands. To ensure the effective distribution of COVID-19 vaccines, the cold chain logistics system plays the most important role. Defined by the WHO, the vaccine cold chain is a system that is used for storing and transporting vaccines at the recommended temperature from manufacturing plants to the locations of administration (Organisation). The research focus has been given to the temperature monitoring and control of the vaccine cold chains. For example, phase change materials (PCMs) are used for cold chain storage equipment (Xiaofeng & Xuelai, 2021; Zhao et al., 2020). Besides, the use of several cutting-edge Industry 4.0 technologies, i.e., radio frequency identification (RFID), internet of things (IoT), 5G, and blockchain in the vaccine cold chain have also been discussed (Bamakan et al., 2021). The combination of RFID and wireless sensor networks (WSN) enables automatically temperature monitoring in the storage and transportation in the vaccine chains (Santos et al., 2015; Zhou & Chakrabartty, 2017). With the help of 5G and IoT, the vaccine cold chain logistics systems can be better monitored and controlled with real-time information of the temperature, warehouse, vehicles, and personnel (Li, 2021).

The sustainable planning of vaccine supply chains and cold chain logistics systems is a complex decision-making problem, which has been investigated by several studies from both economic and environmental perspectives, e.g., CO_2_ emissions (Leng et al., 2020a, 2020b; Saif & Elhedhli, 2016; Yang et al., 2021). Besides, several studies focus on the operational challenges related to vaccine logistics systems, e.g., technology and responsiveness (Dasaklis et al., 2012; Dwivedi et al., 2018; Privett & Gonsalvez, 2014; Yong et al., 2020). From the strategic perspective, the most extensively used techniques for sustainable vaccine supply chain network design are multi-objective programming (MOP) and mixed-integer linear programming (MILP) (Lemmens et al., 2016), which are capable of making both facility location and linkage decisions considering multiple sustainability objectives. de Carvalho et al. (2019) proposed a multi-objective MILP for sustainable vaccine supply chain network design to balance the costs, emissions, and social sustainability based on GDP per capita. To better tackle uncertainty, Sazvar et al. (2021) investigated a robust fuzzy MOP to minimize the costs, pollution, the deviations from the expected social indicator in the network design of an influenza vaccine supply chain. The use of these MOP models yields Pareto optimal solutions, which balance the tradeoff among economic, environmental, and societal objectives. To calculate these solutions, weighing methods and constrained methods have been widely used (Lemmens et al., 2016). Furthermore, recent research has also focused on the use of heuristics and metaheuristics to improve the computational efficiency in sustainable vaccine supply chain design (Duijzer et al., 2018).

From the operational perspective, the transportation planning of the vehicles and the routes has been investigated for vaccine cold chains. In this regard, the time window is an important consideration to ensure responsive vaccine delivery (Ma et al., 2006). Zheng et al. (2013) investigated a capacitated vehicle routing problem (CVRP) with breakdowns for the last-mile vaccine distribution. Petroianu et al. (2021) developed an off-the-shelf route optimization tool for last-mile vaccine logistics planning in Mozambique. During the COVID-19 pandemic, sustainable vaccine logistics operations are of paramount importance to control the disease spread. Gamchi et al. (2021) investigated a bi-objective vehicle routing problem (VRP) for last-mile vaccine logistics considering both economic and social costs, and the social costs were measured by the service level of different priority groups based on the SIR model. Pasha et al. (2020) investigated a two-stage optimization model based on the concept called “Factory-in-a-box”. The model connects both factory planning and logistics distribution to improve resource utilization and the responsiveness to urgent customer needs.

**Fig. 1:**
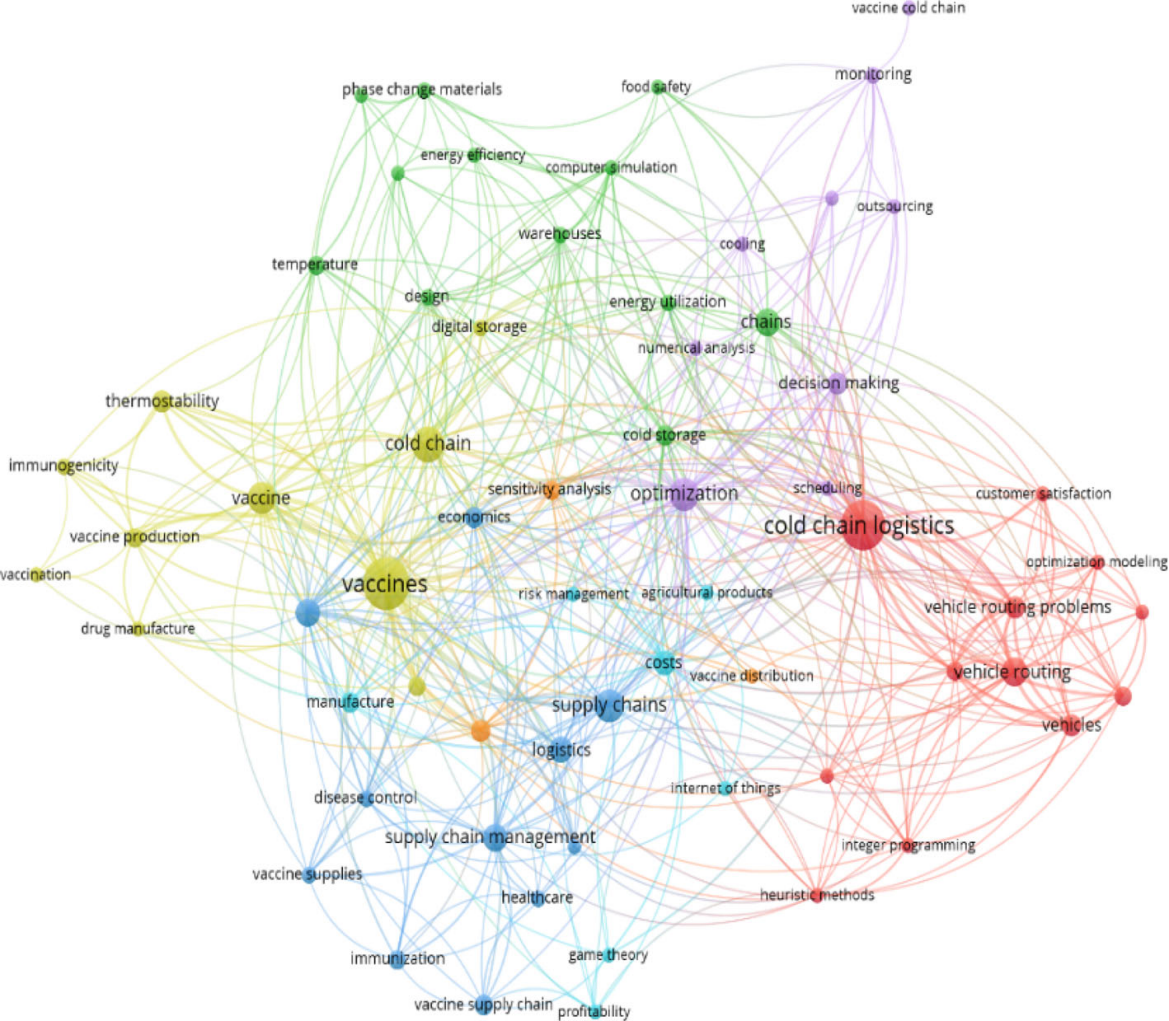
Keyword co-occurrence map of the vaccine supply chain and cold chain logistics

Figure 1 presents the research focus of vaccine supply chains and cold chain logistics through a keyword co-occurrence analysis. The COVID-19 pandemic has led to several new challenges for sustainable vaccine supply chains and cold chain logistics in economic, environmental, and societal dimensions (Alam et al., 2021; Jiang et al., 2021; Klemeš et al., 2021). However, the current methods cannot fully address these new challenges. From the analysis of the relevant research, the following research gaps are identified:

1. The sustainability issues are investigated mainly from the strategic network design level of vaccine supply chains, but the research at the operational level for sustainable last-mile vaccine cold chain logistics operations has not been thoroughly studied.
2. The new challenges for COVID-19 vaccine logistics operations have not been identified and tackled with quantitative analytical methods.
3. From the methodological development perspective, mathematical optimization has been extensively used for improving vaccine cold chain logistics operations. However, there is a lack of combining both optimization models with advanced simulation, e.g., discrete event simulation, in a decision-support approach to better capture the real-world features and provide more robust analytical analyses.

Thus, based on a real-world problem in Norway, this paper aims at filling these gaps by providing a new two-stage decision-support approach to solve the emerging challenges related to sustainable last-mile cold chain logistics operations of COVID-19 vaccines.

## Problem description

In this section, the last-mile cold chain logistics system for COVID-19 vaccine distribution in Norway is introduced, and the main sustainability challenges are identified.

### The COVID-19 vaccine cold chain logistics system in Norway

Norway is a Scandinavian country located in Northern Europe and shares borders with Sweden, Finland, and Russia. The total population of the country is over 5.3 million in 2020 (http://www.norgeshelsa.no/norgeshelsa/, https://www.fhi.no/nettpub/koronavaksinasjonsveilederen-for-kommuner-og-helseforetak/distribusjon-av-koronavaksiner/vaksinasjon-ved-koronapandemi/, https://www.fhi.no/en/news/2021/changes-in-the-vaccine-strategy/). With the inhabitants spreading over 385,207 km^2^, Norway is one of the most sparsely populated countries in Europe. Founded on the principle of universal access, the Norwegian health care system is semi-decentralized. Healthcare policies, regulations, and supervision of the entire healthcare system are centrally controlled by the Directorate of Health and the Norwegian Medicines Agency (NMA) under the Ministry of Health and Care Services, while specialized healthcare is administered by the four Regional Health Authorities (RHAs). The local municipalities solely control primary healthcare services, whereas the public healthcare services are managed at both national and regional levels (https://legemiddelverket.no/english/about-us/the-norwegian-health-care-system-and-pharmaceutical-system).

In Europe, the COVID-19 vaccines are first reviewed for use by the European Medicines Agency (EMA). The EMA performs the 'rolling review' for the research sent by different manufacturers before their vaccines are recommended (https://www.vg.no/spesial/corona/vaksiner/). The EMA’s recommendations are then approved by the European Union (EU) Commission for vaccine use across Europe. Through the European Economic Agreement (EEA), the COVID-19 vaccines from the EU Commission can then be included in the Norwegian Immunization Program (https://www.vg.no/spesial/corona/vaksiner/). Then, the COVID-19 vaccines are purchased by the Norwegian Institute of Public Health (NIPH).

**Fig. 2:**
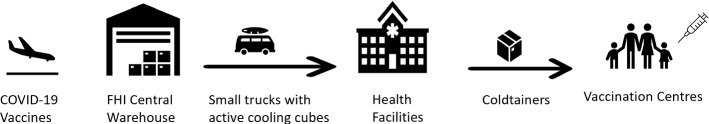
The COVID-19 vaccine cold chain logistics system in Norway (Coronavirus)

Currently, two COVID-19 vaccines have been approved and used in Norway’s Coronavirus Immunization Program, namely, Pfizer/BioNTech and Moderna, where Pfizer/BioNTech’s COVID-19 vaccine takes the predominate share. As shown in Fig. 2, the COVID-19 vaccines that arrived in Norway are received by the NIPH and stored in its central warehouse before direct distribution to several reception centers at the municipality level. The vaccines are shipped directly to the regional health authorities and hospital pharmacies via cold chain logistics systems (https://www.regjeringen.no/en/aktuelt/address-by-the-minister-of-health-and-care-services-to-the-storting-on-18-january-2021-regarding-norwegian-authorities-work-on-vaccines-against-covid-19/id2829245/) to fulfill the ultra-cold temperature requirements. The NIPH controls the weekly distribution of the vaccines to the municipalities with real-time tracking. Direct distribution from the central warehouse to various reception centers is done either by refrigerated trucks or cooling boxes within 0–8 h in Eastern Norway and 24–36 h to the other parts of the country (http://www.norgeshelsa.no/norgeshelsa/, https://www.fhi.no/nettpub/koronavaksinasjonsveilederen-for-kommuner-og-helseforetak/distribusjon-av-koronavaksiner/vaksinasjon-ved-koronapandemi/, https://www.fhi.no/en/news/2021/changes-in-the-vaccine-strategy/).

The current COVID-19 vaccine allocation strategy used by the NIPH is to allocate more vaccine doses to the municipalities with high COVID-19 infection rates. The geographical redistribution based on areas with high infection rates allows for a 20% increase in vaccine doses to Oslo and another four municipalities. Additionally, the distribution will also be affected by the number of inhabitants over 18 in a municipality, which aims at preventing the spread of more contagious variants of the coronavirus in larger urban municipalities (http://www.norgeshelsa.no/norgeshelsa/, https://www.fhi.no/nettpub/koronavaksinasjonsveilederen-for-kommuner-og-helseforetak/distribusjon-av-koronavaksiner/vaksinasjon-ved-koronapandemi/, https://www.fhi.no/en/news/2021/changes-in-the-vaccine-strategy/). The demographic distribution and age proportion of the 11 counties in Norway are presented in “Appendix A” (http://www.norgeshelsa.no/norgeshelsa/, https://www.fhi.no/nettpub/koronavaksinasjonsveilederen-for-kommuner-og-helseforetak/distribusjon-av-koronavaksiner/vaksinasjon-ved-koronapandemi/, https://www.fhi.no/en/news/2021/changes-in-the-vaccine-strategy/).

**Fig. 3:**
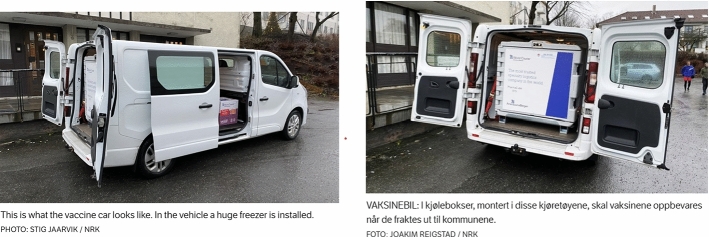
Vaccine Trucks with active cooling cubes (Joakim Reigstad, 2021)

**Fig. 4:**
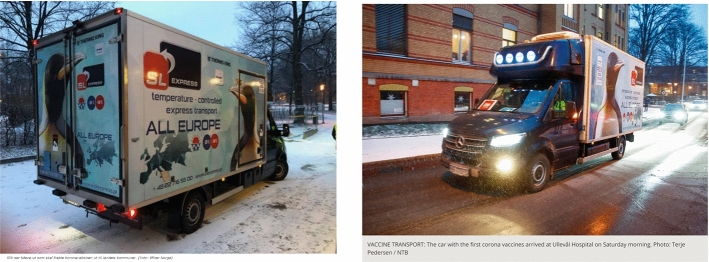
Pfizer/BioNTech vaccine truck (Pedersen, 2020; Pfizer, 2020)

The head of the vaccine department at the NIPH disclosed that the transportation of the COVID-19 vaccines to various municipalities is carried out by third-party logistics (3PL) companies (Knut Jønsrud, 2020). The Moderna vaccine is transported by AmerisourceBergen World Courier, which is a biopharmaceutical logistics company. The deliveries are done with 50 active cooling cubes fitted in small trucks (see Fig. 3) to all parts of Norway except the northern parts of Nordland, where passive cooling containers are used. The Pfizer/BioNTech vaccine is transported by SLexpress refrigerated transport (Knut Jønsrud, 2020) (see Fig. 4). Both 3PL companies use ThermoKing cooling containers and cubes (Joakim Reigstad, 2021).

### Sustainable cold chain logistics challenges for COVID-19 vaccine distribution

The COVID-19 vaccine supply chain in Norway at the early vaccination stage is fully supply-driven and is constrained by the uncertainties of vaccine supplies and deliveries. The COVID-19 vaccines cannot be ordered by the municipalities. Based on the vaccines received from the NIPH at the beginning of each week, the vaccination centers at the municipal level can only plan the administration in a responsive way. However, the number of vaccine doses received is uncertain, which leads to a complex planning problem. The planning of the sustainable last-mile vaccine cold chain logistics system is more challenging due to the requirements on both responsiveness and cold chain infrastructures under high uncertainties. Furthermore, this logistics planning needs to be done in a timely and highly responsive manner based on the number of vaccines received. An improper logistics planning may reduce the system’s sustainability in various dimensions, e.g., higher costs, delayed deliveries, and more carbon emissions. The six most significant challenges for the sustainable last-mile COVID-19 vaccine logistics system are identified as follows:

- *Supply uncertainties* Due to the large demand for COVID-19 vaccines around the world, there is a high degree of uncertainty regarding the vaccine supplies especially at the early stage. The orders cannot be placed according to the number of infections, and the weekly vaccine supply is somewhat uncertain.
- *Time Constraints* Since the COVID-19 vaccines are extremely temperature-sensitive, they must be delivered on time to maintain the effectiveness. Thus, timely delivery is very important to achieve a high level of vaccination coverage and to reduce the waste of vaccine doses.
- *High costs of distribution* A major factor of COVID-19 vaccine distribution is on-time delivery. The associated costs of delivering the vaccines on time at regular intervals to a large number of geographically dispersed communities are very high.
- *Route optimization* The transportation route optimization under high uncertainties in the last-mile vaccine logistics system is of key importance. This has an impact on the expected lead time of deliveries and the associated transport costs.
- *Cold chain requirements* To store and transport the vaccines to local vaccination centers, effective cold chain logistics operations are required. Storage facilities and refrigerated trucks that adhere to the temperature requirements of COVID-19 vaccines need to be in place to ensure the flexibility and viability of vaccine distribution.
- *Environmental impact* A large number of regular small-amount vaccine deliveries to local vaccination centers increase the carbon emissions from the refrigerated trucks, which is one of the major environmental pollutions caused by the last-mile COVID-19 vaccine logistics system.

## Method

In this section, we proposed a two-stage decision-support approach that combines both route optimization and simulation experiments for sustainable last-mile vaccine cold chain logistics operations.

### The two-stage decision-support approach

Optimization and simulation are the two most important analytical tools used in the network design and operational planning of logistics systems. However, they are different techniques having their specific problem domains (Yu et al., 2021). Optimization is prescriptive analytics, which aims at finding the optimal solution through formulating a mathematical model and solving it with an algorithm. Thus, the strength of optimization is the capability of selecting the best solution from a large number of alternatives with the pre-defined rules, namely, objectives and constraints. However, the most significant drawback of optimization is that many real-world characteristics need to be simplified in order to fit the mathematical programming structure and ensure high computational efficiency. To tackle this problem, simulation can be used as a complement to improve the modeling and analysis of real-world problems. On the other hand, simulation is descriptive analytics and can only be used to evaluate the system performance with more realistic settings, but it cannot be used to effectively select the optimal solution from a large number of possible solutions. Table 1 shows the comparison between mathematical optimization and advanced computer-based simulation. To use the strengths of both methods, optimization and simulation can be combined in different ways (Tordecilla et al., 2021). For example, simulation can be first used to estimate some key parameters for the optimization model (Costa et al., 2017; Goodarzian et al., 2021a, 2021b). On the other hand, optimization can be first applied to make key decisions that are then evaluated in a more realistic and comprehensive simulation environment (Sun et al., 2019; Yu et al., 2021).

**Table 1:** Comparison between mathematical optimization and advanced simulation

	Mathematical optimization	Advanced computer-based simulation
Method	Prescriptive analytics	Descriptive analytics
Problem-focused	Problem-solving with a large number of alternatives	Comprehensive performance analysis of limited scenarios
Uncertainty	Parametric randomness	Parametric randomness, dynamic changes in system configuration and policies, and operational uncertainty
Modeling	A highly abstract and simplified system with many assumptions	A realistic system representation with minimum assumptions
Data aggregation	High level	Low level
Analysis	Static	Dynamic
Outputs	Statistically optimum	Practical analytical result

**Fig. 5:**
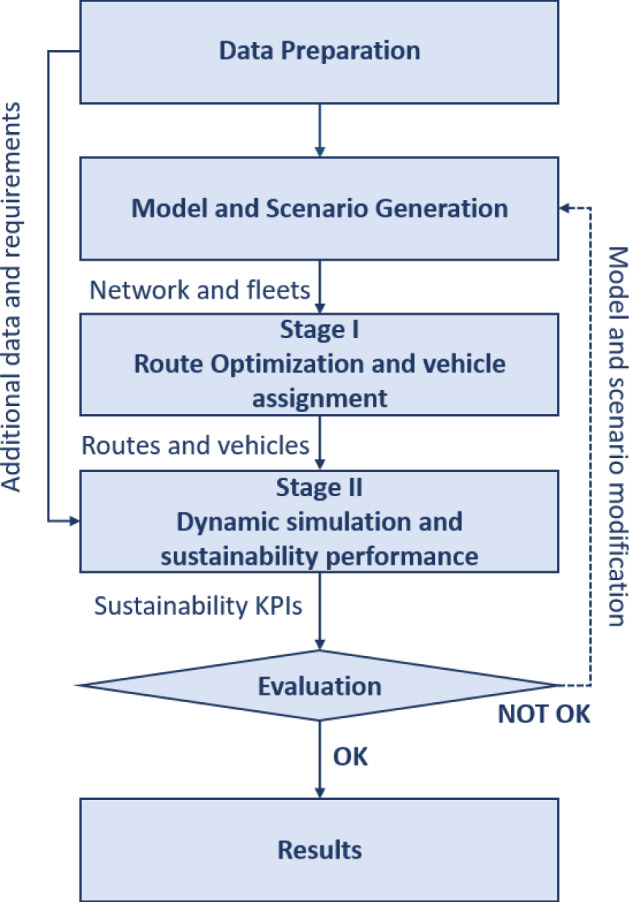
The framework of the two-stage decision-support approach

Figure 5 illustrates the two-stage decision-support approach for sustainable last-mile vaccine cold chain logistics operations. The first step is to prepare the experimental data, based on which the models and scenarios with different logistics network structures, fleet sizes, and fleet composition can be established for optimization and sustainability assessment. In the analytical process, the first stage is the route optimization between the warehouse for COVID-19 vaccines and the vaccination centers, and heterogeneous vehicles are assigned to different routes based on their capacities and maximum travel distances within the time window. Next, these routing and vehicle assignment decisions are inputted into the discrete event simulation model in the second stage, where the performance of the vaccine logistics system is tested under dynamic and realistic conditions. More operational parameters of the last-mile vaccine cold chain logistics system, e.g., facility handing, working time requirements, delayed delivery, stochastic operating time, etc., need to be defined to run the simulation model and yield improved analyses of the real-world problem. In this stage, the economic, environmental, and societal indicators need to be defined to measure the sustainable performance of the vaccine logistics system. The evaluation results need to be compared with the pre-defined benchmark indicators. If the sustainability performance of the vaccine logistics system cannot fulfill the expected requirement, the system needs to be modified with new models and scenarios.

### Route optimization

The transport routes between the vaccine warehouse and the local vaccination centers are first optimized. This is a capacitated vehicle routing problem (CVRP) that has been extensively investigated (Laporte, 2009). Based on Kilby and Shaw (2006), a modified CVRP with heterogamous vehicles is formulated for the route optimization of last-mile vaccine cold chain logistics operations. The set of nodes is defined by $V=\left\{\mathrm{0,1}, \dots ,v\right\}$, where $\left\{0\right\}$ is the warehouse of COVID-19 vaccines and $\left\{1,\dots ,v\right\}$ is the set of vaccination centers at the municipalities. The set and index of vehicles are given by $P$ and $k$, respectively. ${c}_{k}$ is the unit transportation cost by vehicle $k$. ${d}_{ij}$ is the distance between two nodes in $V$. ${q}_{i}$ denotes the demand for COVID-19 vaccines at vaccination center $i$. ${Q}_{k}$ is the capacity of vehicle $k$. ${x}_{ijk}$ is a binary decision variable determining if a route between two nodes is selected with vehicle $k$.

1 $$ {\text{Minimize}} \mathop \sum \limits_{i \in V} \mathop \sum \limits_{j \in V} \mathop \sum \limits_{k \in P} c_{k} d_{ij} x_{ijk} $$

Subject to:

2 $$ \mathop \sum \limits_{i \in V } \mathop \sum \limits_{k \in P} x_{ijk} = 1,\quad \forall j \in V\backslash \left\{ 0 \right\} $$

3 $$ \mathop \sum \limits_{j \in V } \mathop \sum \limits_{k \in P} x_{ijk} = 1,\quad \forall i \in V\backslash \left\{ 0 \right\} $$

4 $$ \mathop \sum \limits_{i \in V} x_{ihk} - \mathop \sum \limits_{j \in V} x_{hjk} = 0,\quad \forall i \in V,k \in P $$

5 $$ \mathop \sum \limits_{i \in V} \mathop \sum \limits_{j \in V} q_{i} x_{ijk} \le Q_{k} ,\quad \forall k \in P $$

6 $$ \mathop \sum \limits_{i \in V} \mathop \sum \limits_{j \in V} d_{ij} x_{ijk} \le D_{k} ,\quad \forall k \in P $$

7 $$ \mathop \sum \limits_{i \in V} \mathop \sum \limits_{j \in V} \mathop \sum \limits_{k \in P} x_{ijk} \le \left| W \right| - 1,\quad \forall W \subseteq V\backslash \left\{ 0 \right\} $$

8 $$ x_{ijk} \in \left\{ {0, 1} \right\},\quad \forall i \in V,\;j \in V,\;k \in P. $$

Equation (1) is the objective function that minimizes the total transportation cost. Constraints (2) and (3) jointly ensure that each vaccination center needs to be served once, which means the demands of all municipalities are be fulfilled. Equation (4) restricts that the COVID-19 vaccines for each vaccination center can only be sent by one vehicle. Constraint (5) sets the capacity constraint. When distributing a small number of COVID-19 vaccines to multiple geographically dispersed municipalities, this constraint can be modified by setting ${q}_{i}$ to 1, and the right-hand side becomes then the maximal number of municipalities a vehicle can visit per tour to maintain high service responsiveness of vaccine distribution. Constraint (6) is the maximal travel distance per tour. Constraint (7) is given to eliminate subtours, and several alternative forms have been formulated for the same purpose, see Ramos et al. (2020). Constraint (8) sets the domain of decision variables. Several CVRP variants set other constraints, e.g., time window (Baldacci et al., 2012), etc. However, this will significantly increase the computational time of the CVRP, which is an NP-hard problem. Thus, these constraints are not considered in this paper, and more practical conditions are tested in the second stage with simulation.

### Simulation experiments

In the second stage, two simulation methods, namely, discrete event simulation and Monte Carlo simulation, are used to model the last-mile vaccine cold chain logistics operations. A discrete event simulation models a system with a set of events organized in a sequential order, which triggers the change of the system’s operations and states dynamically and autonomously (Taha, 2013). Today, with drastically improved computer power, discrete event simulation has been widely used to evaluate complex systems and provide high-quality system visualization. Employing the real-world GIS map over several dynamic periods, the simulation model allows the change of several operational parameters, e.g., transport vehicles, fleet size, vehicle combinations, etc., and it can also be used to perform a set of *what-if* scenarios to measure and evaluate sustainable performance indicators of the last-mile vaccine cold chain logistics system under a dynamic and realistic environment. Compared with mathematical optimization models, a discrete event simulation can better model the real-world uncertainties with stochastic parameters (Yu et al., 2021), e.g., the handling time of COVID-19 vaccines at the warehouses and local vaccination centers, the speed of vehicles, etc. Furthermore, several practical requirements related to the logistics operations for COVID-19 vaccine delivery, e.g., expected lead time of different vaccination centers, the time window of logistics services, dynamic demands, etc., can be properly modeled in the simulation stage. Due to the use of stochastic parameters, the simulation experiment needs to be repetitively performed several times in order to validate the results with different scenario trees. This step is Monte Carlo simulation, which aims, through repetitively running the simulation model, at guaranteeing a high level of stability and confidence of the analytical results. Simply say, it is expected that the simulation results are determined by the model itself but not by the scenario generation procedure of stochastic parameters. In the latter case, the result may change drastically every time the simulation model is run, and the instability may lead to improper managerial decisions. Thus, the stability test is important in the simulation stage. For more details, the research by King and Wallace (2012) and Yu et al. (2020a) can be referred to.

To evaluate the sustainability of the last-mile cold chain logistics system of COVID-19 vaccines, the performance indicators in societal, economic, and environmental dimensions are given in Table 2. The primary objective of the sustainable last-mile vaccine cold chain logistics system in Norway is to effectively distribute the COVID-19 vaccines to a large number of geographically dispersed vaccination centers at the municipality level. Thus, the primary performance indicator is societal sustainability, which is measured by the service level and lead time. The lead time by the customer is the total time taken for the vaccine delivery between the warehouse and the local vaccination centers. In this study, the ordering time at the municipal level is at the beginning of each week. The service level is the ratio of the products delivered on time to the overall number of products shipped. If the COVID-19 vaccines are delivered within the pre-defined time windows, it is considered an on-time delivery. To improve the social sustainability of last-mile vaccine cold chain logistics operations, high responsiveness needs to be achieved by shortening the lead time and maximizing the service level. In addition, transportation costs and CO_2_ emissions are used as measures to evaluate economic and environmental sustainability.

**Table 2:** Performance indicators for sustainable vaccine cold chain logistics operations

Sustainability dimensions	Performance indicators
Societal sustainability	Service level and lead time
Economic sustainability	Transportation costs
Environmental sustainability	CO_2_ emissions

## Case study

In this section, the effectiveness and applicability of the proposed method are illustrated through a real-world case study in Inland County, Norway.

### Studied area and data collection

Inland is the second-largest county located in the eastern part of Norway, which has a population of 371,385 with 46 municipalities divided into ten regions (Fylkeskommune). Based on the vaccine allocation strategy set by the NIPH, it is crucial to choose an urban county with high COVID-19 infection rates and a large population of inhabitants over 18 years old to evaluate the sustainable performance of last-mile vaccine cold chain logistics operations. As shown in “Appendix A”, the population over 18 in Inland County is 301,975, which accounts for 81% of the entire population.

The data includes the structure of the last-mile logistics network for COVID-19 vaccines, operational strategies, geographical location of the FHI’s warehouse and the local vaccination centers, transportation modes, vehicle types and capacities, demands, and the time windows for receiving the COVID-19 vaccines. The data used in this case study was mainly from the NIPH's database, the VG.no Coronavirus live update, and the information of the municipalities. Besides, secondary data was acquired through email interviews and phone calls with respective stakeholders. The transportation modes, vehicle fleet size, and some operational strategies were provided by the vaccine supply department of the NIPH. Besides, some assumptions were also made.

#### Logistics network for COVID-19 vaccine distribution

**Fig. 6:**
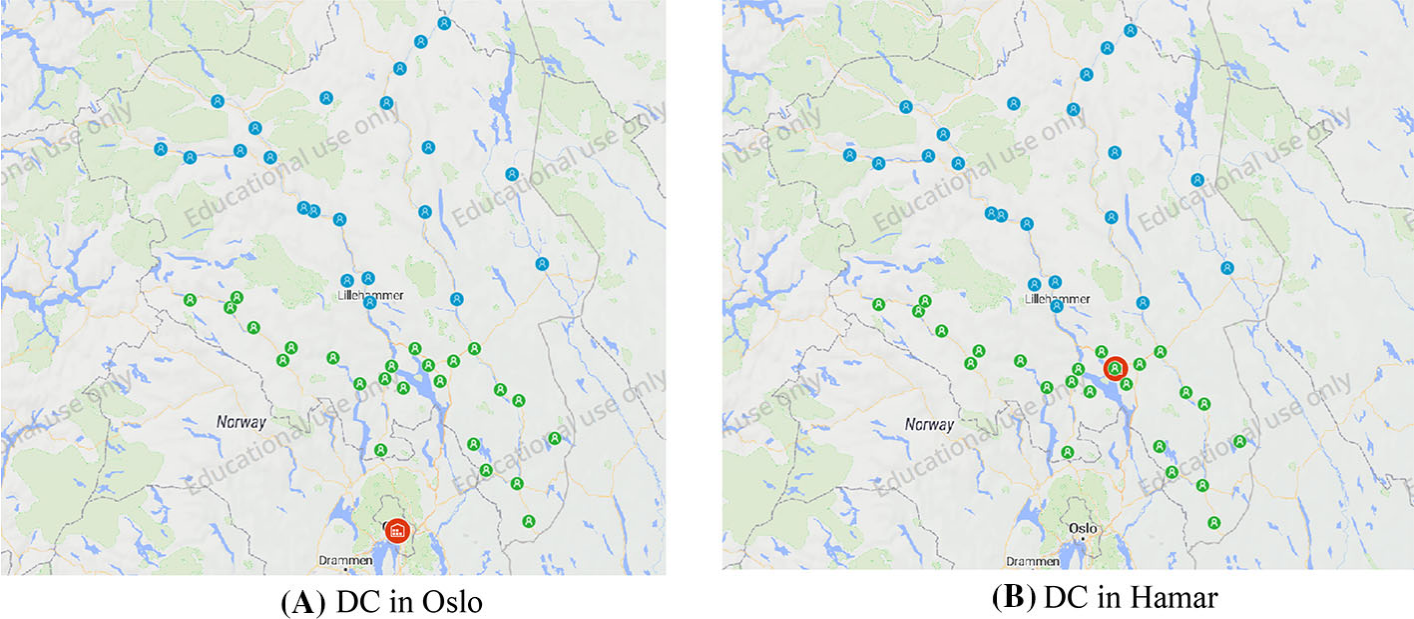
Last-mile cold chain logistics network for COVID-19 vaccine distribution

The customers are the 46 vaccination centers at the municipality level in Inland County. Two network structures for COVID-19 vaccine distribution were considered, as shown in Fig. 6. Considering different network structures and fleet composition, we built three basic models. Models 1 and 2 employ direct transportation of COVID-19 vaccines from the FHI’s central warehouse in Oslo (Knut Jønsrud, 2020). Model 3 uses transshipment via a DC opened in Hamar to improve the efficiency of logistics operations. Two customer groups, (1) *Customer Group 1* and (2) *Customer Group 2* were created. *Customer Group 1* includes the set of municipalities with closer proximity to the DC, while *Customer Group 2* is the vaccination centers that are further away from the DC.

The outbound processing time at the DC was set to follow a uniform distribution between 20 and 30 min. The inbound processing time at local vaccination centers was set to follow a uniform distribution between 10 and 20 min. The shipment of COVID-19 vaccines from the DC to local vaccination centers are performed by three types of vehicles. The shipping policy used was set to less than truckload (LTL) to allow the orders could be shipped no matter if a vehicle is fully loaded or not. The expected lead time (ELT) was prioritized in this case study to ensure high responsiveness of last-mile vaccine cold chain logistics operations. In addition, based on the Norwegian Labour Act, the working hours were set from Monday to Friday, and the shipping needs to be carried out between 6:00 am to 3:00 pm (https://www.arbeidstilsynet.no/en/working-conditions/working-hours/). The sourcing policy for this analysis was set to “fixed sourcing” based on the delivery time to each local vaccination center.

#### Demand

The demand data was obtained from the NIPH’s database within the period between 12/28/2020 and 3/15/2021, see “Appendix B”. The input demand data was set accordingly on a weekly basis with the ELT of one day for *Customer Group 1* and of two days for *Customer Group 2* (http://www.norgeshelsa.no/norgeshelsa/, https://www.fhi.no/nettpub/koronavaksinasjonsveilederen-for-kommuner-og-helseforetak/distribusjon-av-koronavaksiner/vaksinasjon-ved-koronapandemi/, https://www.fhi.no/en/news/2021/changes-in-the-vaccine-strategy/). The backorder policy was set to be “allowed total”. It means that the order is kept pending until the required number of vaccines is available for shipping.

#### Products

The COVID-19 vaccination in Norway is free, so the product selling price is not considered. The unit conversion was set to 600 doses per cubic meter (600 doses/m^3^) in this study.

#### Vehicle types and transportation policies

The COVID-19 vaccine distribution in Norway is carried out by the NIPH using 3PL service providers with 50 active cooling cubes fitted in small trucks (Knut Jønsrud, 2020). In the case study, we considered three types of vehicles, namely, Nissan NV200, Nissan e-NV200 (electric vehicle), and Volkswagen (VW) Transporter. In models 1 and 2, the capacities of the vehicles were set to be 2.1 m^3^ based on the capacity of the active cooling cubes used (https://europe.thermoking.com/wp-content/uploads/2019/07/TK80017_ColdCube_Pharma-07-2020-EN_V1.0.pdf). In model 3, the capacity of VW transporter capacities was set to 6.7 m^3^ (https://www.vg.no/bil/nissan/nv200/spesifikasjoner, https://www.parkers.co.uk/vans-pickups/volkswagen/transporter/2015-dimensions/). The vehicles' speeds were set to be triangular with 40 km/h as the minimum speed, 70 km/h as the maximum speed, and a mode speed of 50 km/h based on the Norwegian traffic rules on speed limit (Nyinorge). The maximum ranges of Nissan NV200 and VW Transporter were set to 1000 km, and the maximum range of the Nissan e-NV200 was set to 300 km (https://www.nissan.no/biler/nye-biler/e-nv200/teknisk-informasjon.html#freeEditorial_contentzone_f70d).

The two customer groups were served by heterogeneous vehicles. Nissan e-NV200 was assigned to transport the vaccines to *Customer Group 1*, and Nissan NV200 was used to serve *Customer Group 2* in models 1 and 2. In model 3, *Customer Group 1* was served by Nissan NV 200, and *Customer Group 2* was served by VW Transporter. The transportation cost was calculated using "distance-based with fixed cost policy". These three types of vehicles are considered in the same category as small trucks. The fixed cost was derived from the drivers' salary and was set to a constant value of 1500 NOK (Leng et al., 2020a, https://www.ssb.no/en/statbank/table/11421/). The variable cost is determined by fuel and electricity consumption, which is directly proportional to travel distance. The relevant parameters were estimated based on the data from Statistics Norway (https://www.ssb.no/en/statbank/table/11421/), as shown in Table 3. The CO_2_ emissions from Nissan NV200 and VW Transporter were set to 131 g/km and 159 g/km, respectively (https://www.vg.no/bil/nissan/nv200/spesifikasjoner, https://www.nextgreencar.com/emissions/make-model/vw/transporter%20kombi%20crew%20van%20t6.1/), and the electric vehicle was assumed to be zero-emission (tank-to-wheel) (https://www.nissan.no/biler/nye-biler/e-nv200/teknisk-informasjon.html#freeEditorial_contentzone_f70d). Besides, since the shipping of COVID-19 vaccines was only performed in one or two days per week, the cost of fleet maintenance, the cost of vehicles, and other transportation policies were not taken into account in this experiment.

**Table 3:** Vehicle parameters

Parameters	Description	Values	Units
Vehicle capacity	Nissan NV200	2.1	m^3^
	Nissan e-NV200	2.1	m^3^
	VW transporter	6.7	m^3^
Vehicle speed	Variable speed	Triangular (40;70;50)	km/h
Vehicle range	Nissan NV200	1000	km
	Nissan e-NV200	300	km
	VW transporter	1000	km
CO_2_ emissions	Nissan NV200	131	g/km
	Nissan e-NV200	0	g/km
	VW transporter	159	g/km
Transportation costs	Variable cost (e-NV200/NV200/VW Transporter)	6/8/10	NOK/km
	Fixed cost	1500	NOK/stop
Expected lead time (ELT)	Customer Group 1	1	Day
	Customer Group 2	2	
Processing times	Inbound shipment	Uniform(10;20)	Minute
	Outbound shipment	Uniform(20;30)	Minute
Shipping	Shipping time	Monday to Friday 6:00 am to 3:00 pm	Day
Period	Experiment duration	12/28/2020–3/15/2021	Day

### Experiments

The experiments were performed for the duration between 12/28/2020 and 3/15/2021 by using a professional optimization-simulation software package called anyLogistix, which is a cutting-edge integrated solution incorporating both mathematical optimization models and multiple simulation methods for decision support and performance analysis of logistics systems and supply chains (https://www.anylogistix.com/alx-features/, Ivanov, 2019). The two-stage experiments were established in the transportation optimization (TO) module and the simulation (SIM) module. In the TO module, the optimal routing and vehicle assignment decisions can be calculated by the built-in CPLEX optimization solver. The models created in the TO are exported into the SIM, and the simulation experiments are then run with the results obtained from the TO experiments. The SIM is established based on the AnyLogic simulation package, which can effectively perform discrete even simulation and Monte Carlo simulation (Ivanov, 2020).

#### Transportation optimization (TO)

**Fig. 7:**
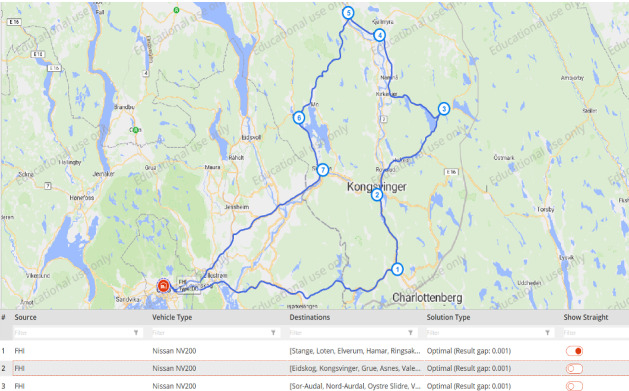
An example of the selected route for COVID-19 vaccine distribution

The first sets of experiments were performed in the TO, where three main models were developed to evaluate different network structures and transport vehicles. The TO experiments optimize the routing decisions for different types of vehicles. In Models 1 and 3, the DC is located in Oslo, which is the actual warehouse of the NIPH. In Model 2*,* on the other hand, a new DC is opened in Hamar. The DC location was selected by simply using the greenfield analysis (GFA) in anyLogistix, which is a center-of-gravity analysis method based on the real-world GIS and road networks. This new DC is much closer to local vaccination centers, which has significant impacts on the transportation costs, responsiveness, and CO_2_ emissions. Due to the geographical dispersion of the vaccination centers in Inland County, the route capacity was set to 10 vaccination centers per trip to ensure high responsiveness and service level to respective customer segments. Similarly, the travel segment and returning segment limits are constrained by the range of vehicles, customer locations, and service level. In models 1 and 2, the optimal solution yielded five routes from the respective DCs to *Customer Group 1* with Nissan e-NV200 and three routes to *Customer Group 2* with Nissan NV200. In Model 3, the optimal solution yielded three routes to *Customer Group 1* using Nissan NV200 and five routes to *Customer Group 2* with VW Transporter. Figure 7 shows an example of the optimal route generated by the TO.

#### Simulation experiments

The optimal routes (milk runs) obtained by the TO experiments are then exported into the SIM module. In the simulation experiments, different ELTs were defined for each model. In Models 1 and 3, the ELT for *Customer Group 1* was set to one day, and it was set to two days for *Customer Group 2.* The ELTs for both customer groups in Model 2 were set to one day. This is because the DC in Model 2 is much closer to the local vaccination centers at the municipality level. The simulation experiments aim to evaluate the economic, environmental, and societal sustainability indicators of the COVID-19 vaccine logistics system in a realistic environment.

**Fig. 8:**
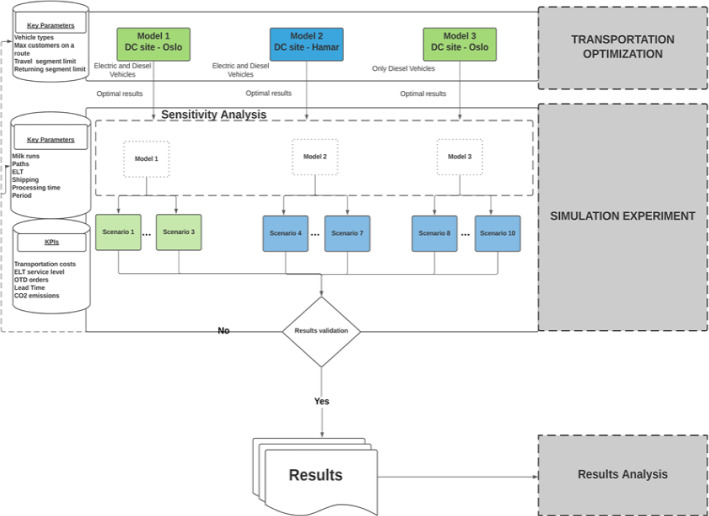
The setting of the two-stage optimization-simulation experiments

As shown in Table 4, by varying the fleet size and fleet composition, ten test scenarios were generated based on Models 1, 2, and 3 to provide insights into the sustainable last-mile logistics operations for COVID-19 vaccine distribution. Due to the use of several stochastic parameters to simulate the real-world environment and to estimate the impacts of uncertainty, the reliability of the simulation results is extremely important. Thus, the simulation experiments of each scenario were validated via 30 replications to yield reliable analytical results with a high level of confidence. Figure 8 illustrates the setting of the two-stage experiments and the required data in each stage.

**Table 4:** Test scenarios in the simulation experiments

Model	Scenario	Fleet size	Fleet composition		
			Nissan e-NV200	Nissan NV200	VM Transporter
Model 1	1	3	1	2	–
	2	5	3	2	–
	3	6	4	2	–
Model 2	4	3	1	2	–
	5	5	3	2	–
	6	6	4	2	–
	7	7	4	3	–
Model 3	8	3	–	1	2
	9	5	–	2	3
	10	6	–	3	3

## Results and discussions

The experimental results are first given, and managerial implications related to the sustainable last-mile vaccine cold chain logistics operations are then discussed.

### Experimental results

Table 5 presents the performance indicators of economic, environmental, and societal sustainability in the 10 test scenarios. Herein, the ELT service level indicates the percentage of the on-time delivery of COVID-19 vaccines at the local vaccination centers.

**Table 5:** Experimental results

Scenario	Fleet size	Economic indicator	Environmental indicator	Societal indicator		
		Costs (NOK)	CO_2_ emissions (g)	ELT service level	Lead time	
					Mean	Max
1	3	567,802	3,379,831	0.423	1.229	4.369
2	5	605,802	3,432,275	0.946	0.67	1.554
3	6	601,099	3,379,831	1	0.628	1.486
4	3	448,227	227,003	0.511	0.904	3.329
5	5	476,930	227,003	0.921	0.541	1.377
6	6	476,930	227,003	0.928	0.518	1.378
7	7	476,930	227,003	1	0.477	0.857
8	3	705,387	8,326,851	0.657	1.191	2.751
9	5	726,565	8,521,879	0.969	0.73	1.62
10	6	726,565	8,521,879	1	0.652	1.616

#### Model 1

**Fig. 9:**
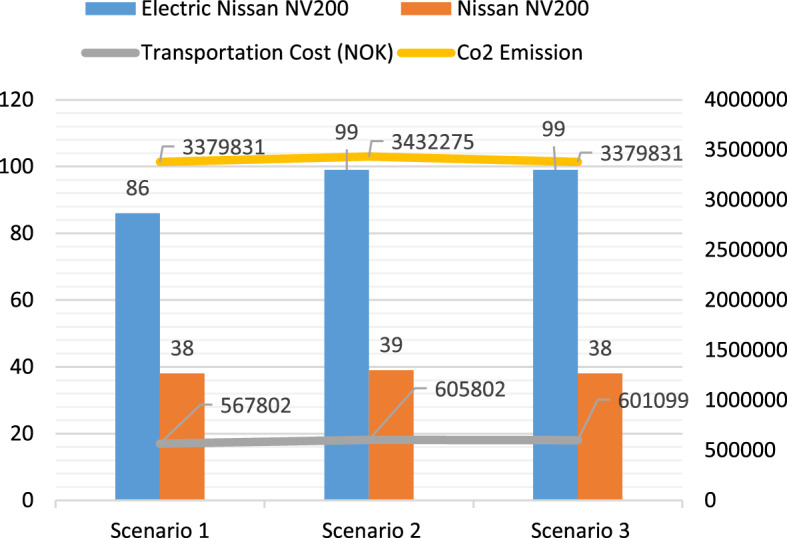
The transportation cost, CO_2_ emissions, and the total trips performed by Nissan NV200 and Nissan e-NV200 in Model 1

In Model 1, the fleet size is varied in each scenario by keeping a constant number of Nissan NV200, while increasing the number of the electric Nissan NV200 vehicle. In scenario 1, all KPIs have low performance due to the small fleet size, and only 42.3% of the COVID-19 vaccines can be delivered to the local vaccination centers on time. Increasing the fleet size from 3 to 5, the service level can be increased to 94.6%, and the average lead time can be reduced from 1.229 to 0.67 days. With an increasing fleet size in scenario 3, the responsiveness of the logistics system is improved, and all municipalities can receive the vaccines within their ELTs. Figure 9 shows the comparison of the total transportation cost, CO_2_ emissions, and total trips performed by the two types of vehicles. The transportation cost and CO_2_ emissions increase from scenario 1 to scenario 2 since more trips are performed to improve the service level. In scenario 3, the total transportation cost and CO_2_ emissions have small reductions compared with that in scenario 2.

#### Model 2

**Fig. 10:**
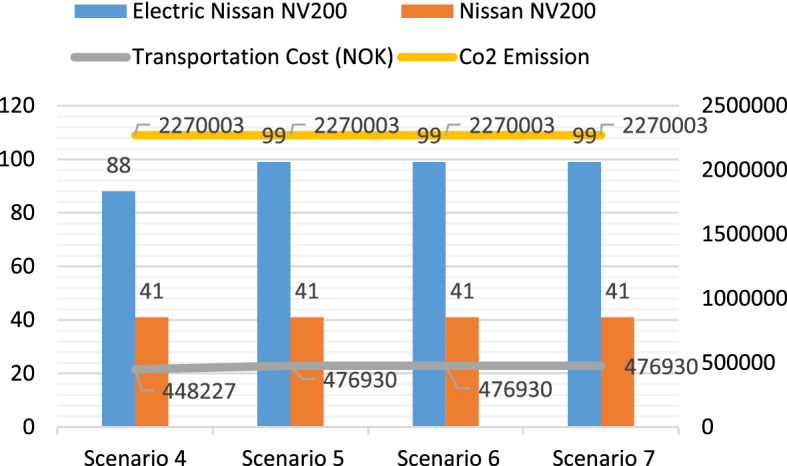
The transportation cost, CO_2_ emissions, and the total trips performed by Nissan NV200 and Nissan e-NV200 in Model 2

In scenario 4, only 51.1% of the COVID-19 vaccines can be distributed to the local vaccination centers within their ELTs. By adding two more electric vehicles to the fleet, the service level can be increased to 92.1%. When one more Nissan e-NV200 is added in scenario 6, the service level can be slightly improved, and the other performance indicators are similar to the previous scenario. Besides, it is also noted that even though the service level and the mean lead time are improved in this scenario, the maximal lead time is 1.378 days. With the fleet size increasing to 7, all the customer demands can be fulfilled within one day. In this scenario, the mean lead time is 0.477 days, and the maximal lead time becomes 0.857 days. Figure 10 shows the transportation cost, CO_2_ emissions, and the total number of trips performed by each type of vehicle in Model 2. This research only considers the cost and CO_2_ emissions related to the last-mile delivery of the COVID-19 vaccines, and both are calculated based on the distances and tours. Thus, increasing the fleet size does not necessarily cause an increase in the transportation cost and CO_2_ emissions if the fleets are not fully utilized. However, the service level and lead time can be improved by a large fleet size since more trips can be performed simultaneously in high-demand periods.

#### Model 3

**Fig. 11:**
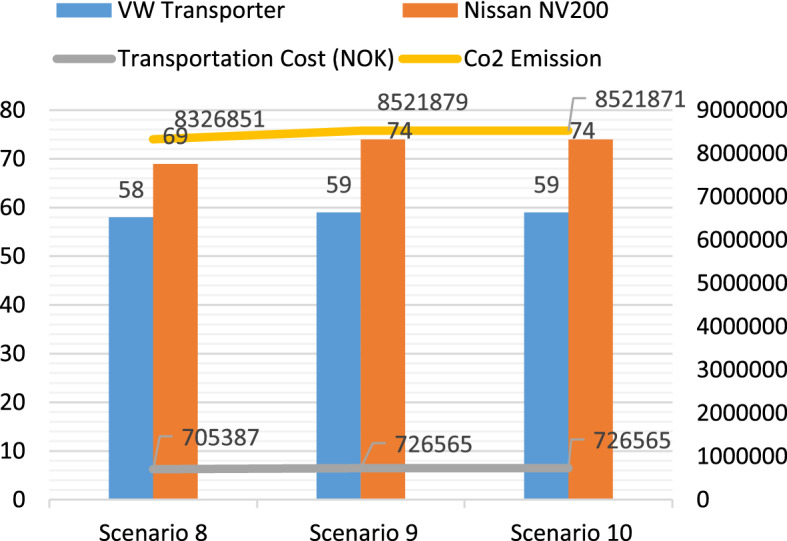
The transportation cost, CO_2_ emissions, and the total trips performed by Nissan NV200 and Nissan e-NV200 in Model 3

With one Nissan NV200 and two VW transporters, scenario 8 yields the lowest transportation cost and CO_2_ emissions in Model 3, and 65.7% of the COVID-19 vaccines can be delivered within the ELTs. In scenario 9, the service level reaches 97% by increasing the fleet size from 3 to 5, and the maximum lead time is reduced from 2.75 to 1.62 days. By adding one more Nissan NV200 in scenario 10, the service level can be improved to 100%. The mean lead time and the maximum lead time are 0.652 days and 1.616 days, respectively. Figure 11 presents the relevant information of scenarios 8, 9, and 10.

### Comparative analysis and discussions

**Fig. 12:**
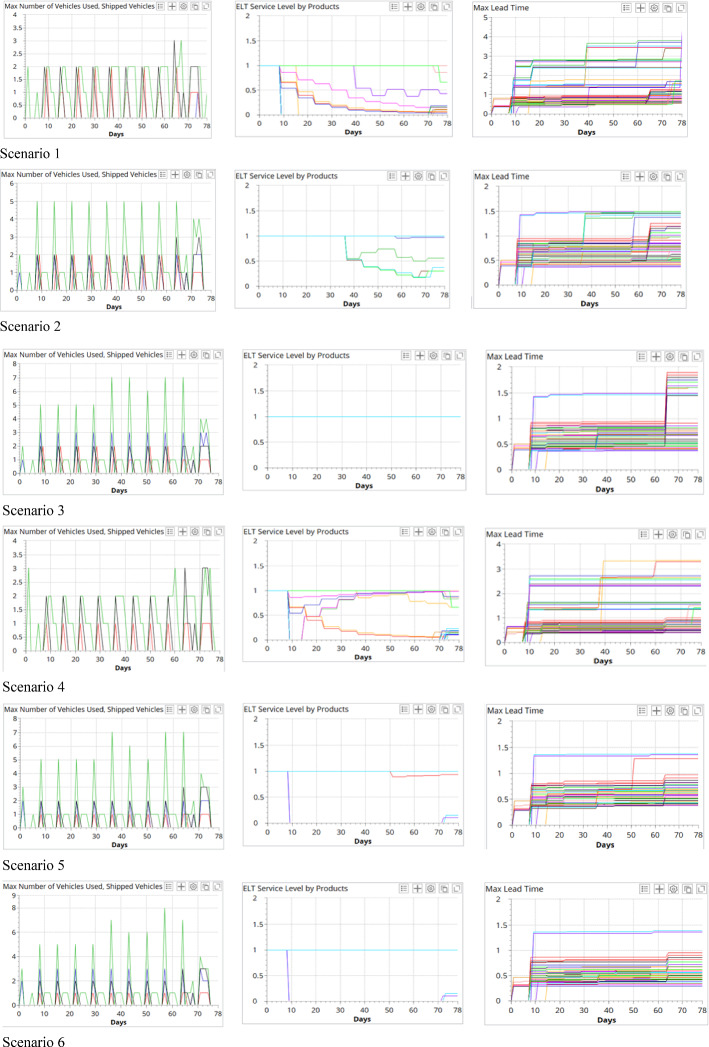

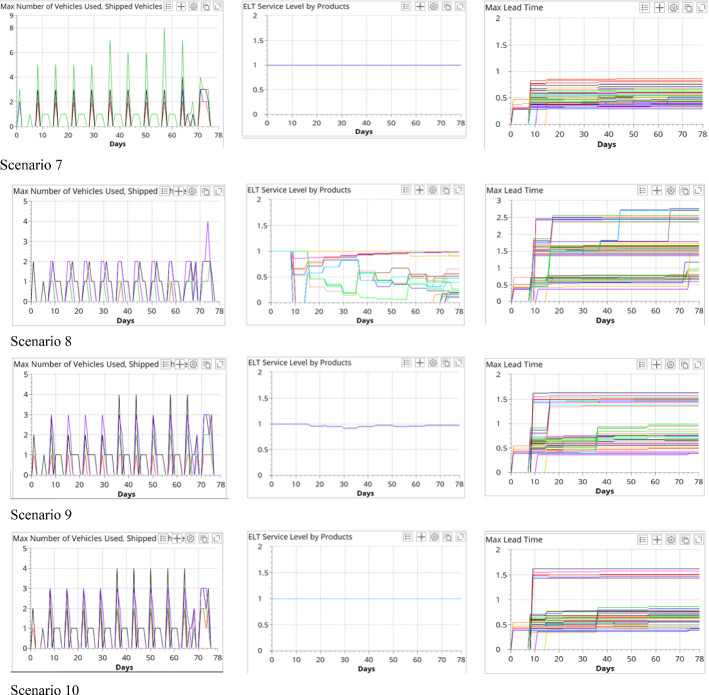
Comparison of the vehicle usage, service level, and maximal lead time of the test scenarios

In this section, a comparative analysis is conducted for all scenarios. Figure 12 compares the number of total and type-specified vehicle usage in each period, the service level of on-time vaccine delivery, and the maximum lead time for all the vaccination centers in the 10 test scenarios. As shown, the economic, environmental, and societal sustainability indicators are affected by the logistics network structure, the types of vehicles employed, and the number of different vehicles in the fleet.

#### Societal sustainability

The societal sustainability performance is evaluated by the responsiveness and service level of the last-mile vaccine cold chain logistics operations. During the pandemic, distributing the right quantity and quality of COVID-19 vaccines to local vaccination centers in a highly responsive manner can help to better plan the local vaccination and minimize the wastage of vaccines. The responsiveness is measured by the service level of on-time delivery. When the DC is located in Oslo, the ELT for *Customer Group 1* is 1 day, and the ELT for *Customer Group 2* is 2 days. When the DC is located in Hamer, the ELTs for all customers are 1 day.

It is observed that, with the same network structure and the same types of vehicles, increasing the fleet size will generally improve the service level. For example, in Model 1, when adding three more Nissan e-NV 2000 to the fleet in scenario 1, the service level can be increased from 42 to 94% in scenario 2, and the maximal lead time can also be reduced by 66%. This is because more trips can be performed simultaneously to improve the service level, as shown in the figure. Besides, compared with scenario 3, the reduced service level in scenario 2 is caused by the delay of the vaccine delivery to *Customer Group 1*.

Next, we compared the results of the scenarios from both Models 1 and 3, which shared the same logistics network structure. The service levels vary drastically when the fleet size equals 3. This reveals the impacts of the type of vehicles on the service level in a last-mile vaccine cold chain logistics system. In Model 2, due to the closer proximity from the DC to the local vaccination centers, a shorter lead time can be achieved with the same fleet, and this reveals the impact of logistics network structures on the service level of the vaccine delivery. However, in this model, due to the more stringent ELT requirement, one more vehicle is needed in scenario 7 to achieve 100% on-time vaccine delivery.

#### Economic performance

**Fig. 13:**
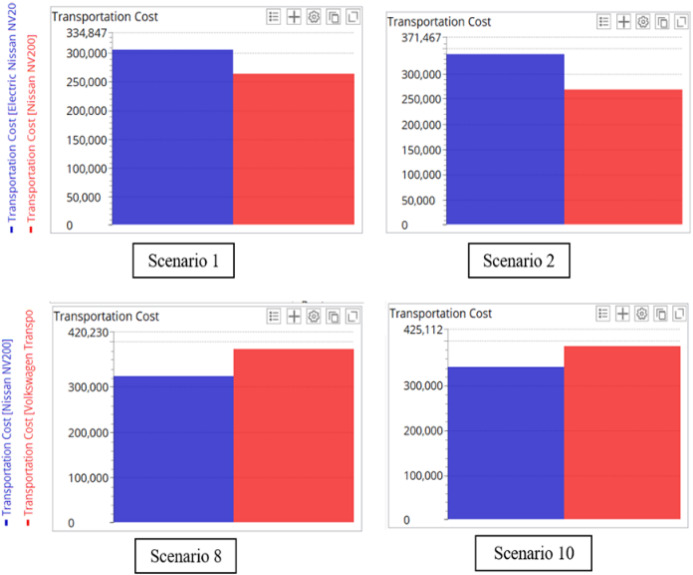
Comparison of the cost components in the four selected scenarios

The transportation costs of the test scenarios in the three models were compared. Due to the advantage brought by the DC location, the scenarios in Model 2 have much lower transportation costs. When comparing Model 1 with Model 3, it is noted that, with the same fleet size, the transportation costs in Model 1 are significantly lower than those in Model 3, even though both models use the same DC. The difference in the transportation costs between these two models is caused by the types of transport vehicles used for COVID-19 vaccine distribution. Figure 13 shows the cost components of the four selected scenarios in Models 1 and 3*.* Even though using VW Transporter can improve the service level particularly in scenarios with small fleet sizes, it will incur higher transportation costs.

#### Environmental performance

Since the CO_2_ emissions from the electric vehicles were set to 0, the CO_2_ emissions were only contributed by Nissan NV200 and VM transporter in the experiments. It is first observed that, even though Model 1 and Model 2 use the same types of transport vehicles (Electric Nissan NV200 and Nissan NV200), the CO_2_ emissions are by no means identical. With a new DC opened in Hamer, the CO_2_ emissions for the last-mile delivery of COVID-19 vaccines can be dramatically reduced. Another interesting observation is found when Model 1 and Model 3 are compared. Even though these two models share the same DC in Oslo, the vaccine distribution by the same-sized fleets in Model 3 yields approximately 148% more CO_2_ emissions than that in Model 1. This notable increase is due to the different types of vehicles used in each model. In Model 1, both electric vehicles and gasoline vehicles are used, so the short-distance deliveries to *Customer Group 1* are primarily performed by electric vehicles. However, in Model 3, only gasoline vehicles are used, and this leads to a significant increase in the total CO_2_ emissions of the logistics system.

### Managerial implications

The effective and sustainable last-mile vaccine cold chain logistics operations rely on appropriate decision-making, which may yield significant impacts on whether the vaccines can be delivered on time and in the right conditions. The results from this study highlight the impacts of the customer distribution, network structure, fleet size, and fleet composition on the societal, economic, and environmental sustainability performances of the last-mile logistics system for COVID-19 vaccines. Based on the analysis, five generic managerial implications are summarized as follows:

- *Insight 1* The network structure of a vaccine cold chain logistics system, i.e., warehouse location and vehicle routing, etc., can drastically affect the service level, lead time, transportation cost, and CO_2_ emissions. In this regard, opening temporary facilities or using temporary warehousing services is a way to ensure sustainable logistics operations when serving a large number of geographically dispersed customers.
- *Insight 2* The fleet size affects sustainable cold chain logistics operations. In general, the service level and lead time can be improved by increasing the fleet size to simultaneously perform more trips, which is an effective way to ensure all customer demands can be fulfilled within their ELTs. From the operational perspective, however, the impacts from fleet size on the transportation cost and CO_2_ emissions are relatively insignificant.
- *Insight 3* The use of different types of transport vehicles directly affects the service level, transportation cost, and CO_2_ emissions, especially when the fleet size is small and insufficient to provide on-time vaccine delivery to all customers, so using the right types of vehicles, in this case, may improve the service level. However, this may also lead to an increase in both transportation costs and CO_2_ emissions.
- *Insight 4* The use of electric vehicles can significantly reduce CO_2_ emissions, while at the same time, maintaining cost-effectiveness. However, these benefits can be achieved only when the electric vehicles are used to serve the customers within a short radius. Otherwise, their battery limits may lead to reduced service levels and high transportation costs.
- *Insight 5* The sustainable performance indicators in the three dimensions, i.e., service level, transportation cost, and CO_2_ emissions, may be in conflict with each other in many cases. Thus, the interactions and impacts of the network structure, fleet size, and fleet composition need to be holistically considered in the last-mile logistics operations for COVID-19 vaccines.

## Conclusion

Today, mass vaccination is undergoing all over the world to control the spread of the pandemic. However, due to several influencing factors, i.e., supply uncertainty, temperature requirements, cold chain logistics infrastructures, resource constraints, and dynamically changed vaccine allocation policy, the planning and operation of a sustainable vaccine logistics system is a complex problem. With a focus on the last-mile COVID-19 vaccine logistics operations in Norway, this paper discusses six main challenges and presents a two-stage decision-support approach for sustainable last-mile vaccine cold chain logistics planning. In the first stage, a CVRP is used for route optimization based on the types of vehicles. A comprehensive simulation model combining both discrete event simulation and Monte Carlo simulation is then developed in the second stage to holistically measure and evaluate the sustainability indicators, i.e., service level, lead time, transportation cost, and CO_2_ emissions. The effectiveness and applicability of the proposed method are shown through a real-world case study in Inland County, Norway, and five implications are generalized to show a better understanding of sustainable last-mile vaccine cold chain logistics planning and operations.

The contributions of this research can be summarized as follows:

- Combining both optimization models and advanced simulation methods, a new two-stage decision-support approach is developed to improve the planning of sustainable last-mile vaccine cold chain logistics operations.
- The applicability and effectiveness of the proposed approach are illustrated with a real-world case study in Norway.
- The experimental results illustrate that the logistics network structure, fleet size, and fleet composition may yield significant impacts on the sustainable performance of a vaccine cold chain logistics system.
- The research generalizes five managerial insights that may help logistics managers with a better understanding of the interactions between the key influencing factors.

In addition, from the practical perspective, these managerial insights may help decision-makers with the selection of 3PL service providers. Moreover, they may also help the logistics companies to better plan and manage their resources in vaccine delivery. Nevertheless, the current research has several limitations:

- Only four sustainable performance indicators are considered in this research, but more measurement indicators, e.g., resilience (Goodarzian et al., 2021a), reliability (Tirkolaee et al., 2020b), etc., may be needed in the decision-support system to obtain a holistic analysis of the COVID-19 vaccine logistics system.
- Although anyLogistix offers an off-the-shelf solution that integrates both optimization and simulation to quickly and effectively analyze vaccine cold chain logistics systems, it suffers from the model’s flexibility and computational efficiency issues when the number of customers is extremely large. For example, the computational time of the CVRP may become very long when the problem size increases drastically. Besides, the comparison with the solution obtained from state-of-the-art algorithms for CVRP as well as other VRP variants is of importance.
- Mass vaccination will lead to increased medical waste generation (Klemeš et al., 2021). However, in this research, the reverse logistics problems for medical waste management (Babaee Tirkolaee & Aydın, 2021; Tirkolaee et al., 2021; Yu et al., 2020b) related to mass vaccination is not taken into account.
- The emerging technologies in Industry 4.0/5.0 may yield significant impacts on sustainable logistics planning and operations (Sun et al., 2021), but these impacts are not considered in the current research.

Therefore, future research is needed to tackle the above limitations by including new sustainable performance indications (Tirkolaee et al., 2020a), developing powerful algorithms for route optimization (de Armas et al., 2019), considering the reverse logistics challenges related to mass vaccination, and evaluating the potential impacts brought by disruptive technologies (Sun et al., 2021). Besides, since the cold chain logistics infrastructures and customer dispersions are by no means identical in different countries and different regions, a comparative study between countries is thus a promising research direction to better understand the impacts of these key influencing factors in last-mile vaccine cold chain logistics operations.

## Data Availability

The datasets generated during and/or analyzed during the current study are available from the corresponding author on reasonable request.

## Appendices

“Appendices A and B” provide supplementary data for the case study, and they can be accessed on: https://uitno.box.com/s/xyqdcnfywr01dktkbzo0htbwsvj2ufg2.
